# Characterisation of the active/de-active transition of mitochondrial complex I^☆^

**DOI:** 10.1016/j.bbabio.2014.02.018

**Published:** 2014-07

**Authors:** Marion Babot, Amanda Birch, Paola Labarbuta, Alexander Galkin

**Affiliations:** Queen's University Belfast, School of Biological Sciences, Medical Biology Centre, 97 Lisburn Road, Belfast BT9 7BL, UK

**Keywords:** A/D, active/de-active transition, BN-PAGE, blue native polyacrylamide gel electrophoresis, DIGE, difference gel electrophoresis, DTNB, 5,5′-dithiobis-(2-nitrobenzoic acid), EEDQ, *N*-ethoxycarbonyl-2-ethoxy-1,2-dihydroquinoline, EMCS, *N*-ε-maleimidocaproyl-oxysuccinimide ester, GSH/GSSG, reduced/oxidised glutathione, HAR, hexaammineruthenium, hrCN-PAGE, high resolution clear native polyacrylamide gel electrophoresis, I/R, ischemia/reperfusion, NADH, dihydronicotinamide adenine dinucleotide, NEM, *N*-ethylmaleimide, NHS, *N*-hydroxysuccinimide, NO, nitric oxide, Q, ubiquinone, RNS, reactive nitrogen species, ROS, reactive oxygen species, SDS-PAGE, sodium dodecyl sulphate polyacrylamide gel electrophoresis, SMP, submitochondrial particles, SPDP, N-succinimidyl 3-(2-pyridyldithio)-propionate, Mitochondrial complex I, A/D transition, Conformational change, Ischaemia/reperfusion, Thiol modification

## Abstract

Oxidation of NADH in the mitochondrial matrix of aerobic cells is catalysed by mitochondrial complex I. The regulation of this mitochondrial enzyme is not completely understood. An interesting characteristic of complex I from some organisms is the ability to adopt two distinct states: the so-called catalytically active (A) and the de-active, dormant state (D). The A-form in situ can undergo de-activation when the activity of the respiratory chain is limited (i.e. in the absence of oxygen).

The mechanisms and driving force behind the A/D transition of the enzyme are currently unknown, but several subunits are most likely involved in the conformational rearrangements: the accessory subunit 39 kDa (NDUFA9) and the mitochondrially encoded subunits, ND3 and ND1. These three subunits are located in the region of the quinone binding site.

The A/D transition could represent an intrinsic mechanism which provides a fast response of the mitochondrial respiratory chain to oxygen deprivation. The physiological role of the accumulation of the D-form in anoxia is most probably to protect mitochondria from ROS generation due to the rapid burst of respiration following reoxygenation. The de-activation rate varies in different tissues and can be modulated by the temperature, the presence of free fatty acids and divalent cations, the NAD^+^/NADH ratio in the matrix, the presence of nitric oxide and oxygen availability.

Cysteine-39 of the ND3 subunit, exposed in the D-form, is susceptible to covalent modification by nitrosothiols, ROS and RNS. The D-form in situ could react with natural effectors in mitochondria or with pharmacological agents. Therefore the modulation of the re-activation rate of complex I could be a way to ameliorate the ischaemia/reperfusion damage. This article is part of a Special Issue entitled: 18th European Bioenergetic Conference. Guest Editors: Manuela Pereira and Miguel Teixeira.

## Highlights

• The potential mechanism of complex I A/D transition is discussed.
• An —SH group exposed in the D-form is susceptible to covalent modification.
• The role of A/D transition in tissue response to ischaemia is proposed.

## Graphical abstract

**Figure:**
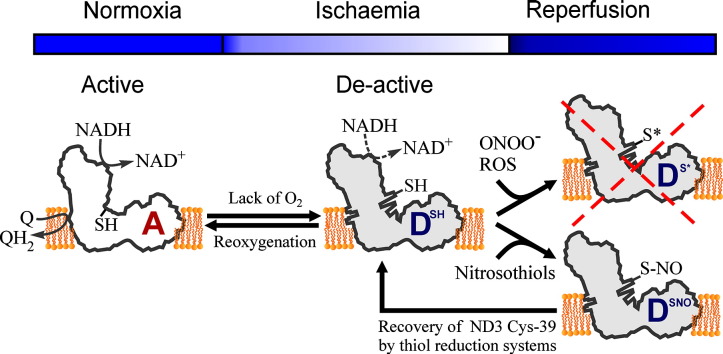

## 1 Introduction

Complex I or NADH:ubiquinone oxidoreductase is a key enzyme of respiration in eukaryotes. In mitochondria, this enzyme is responsible for the oxidation of NADH produced through glycolysis, the Krebs cycle and β-oxidation of fatty acids. Complex I reduces ubiquinone (Q) and transports protons across the inner membrane, contributing to the proton-motive force [1,2]. The enzyme is probably a major contributor to the production of reactive oxygen species in mitochondria [3–5]. The catalytic properties of mitochondrial complex I oxidoreductase are not simple (for a review see [6]). In addition, mitochondrial complex I from mammals and some other species can adopt two catalytically and structurally different states, the so-called active (A) and de-active, dormant (D) conformation.

**Fig. 1:**
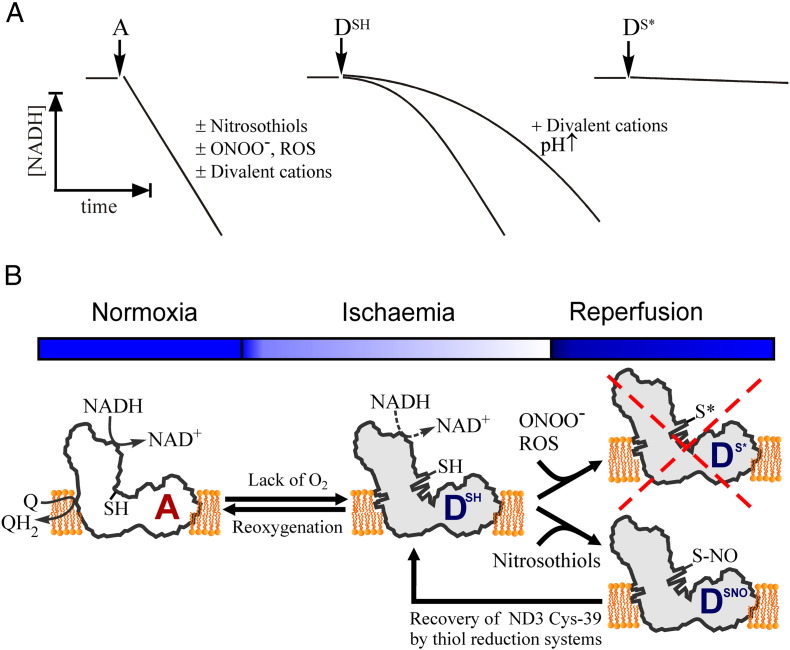
Scheme illustrating the functional and structural aspects of the A/D transition of mitochondrial complex I. A. Time-course of the NADH:ubiquinone (Q) oxidoreductase reaction catalysed by the active (A), deactive (D^SH^) and irreversibly modified (D^S⁎^) forms of the enzyme curves. The A-form of the enzyme catalyses the fast physiological NADH:Q oxidoreductase reaction with a linear rate insensitive to cysteine-modifying reagents such as nitrosothiols, peroxynitrite or ROS. NADH:Q oxidoreductase reaction catalysed by the D-form of the enzyme (D^SH^) proceeds with a lag phase when the D→A conversion takes place. The lag phase is significantly prolonged in the presence of divalent cations or at alkaline pH. Covalent modification of the Cys-39 residue of subunit ND3 of the D-form prevents complex I from undergoing turnover-dependent reactivation and irreversibly inhibits the enzyme (D^S⁎^). B. Possible sequence of events in conditions of ischaemia or lack of oxygen. If oxygen is absent the A-form (A) spontaneously converts to the D-form (D^SH^), which can be re-activated back in the case of reoxygenation (given substrate ubiquinone availability). Depending on the particular conditions in situ the ND3 thiol group residue can be reversibly S-nitrosated by nitrosothiols (D^SNO^) or irreversibly oxidised by peroxynitrite or ROS (D^S⁎^). In the latter case the enzyme is irreversibly inhibited making this the initial step of mitochondrial damage in I/R. S-nitrosated enzyme (D^SNO^) can be reduced by mitochondrial glutathione and thioredoxin [90,117] therefore further delaying the re-activation of the complex I at the early stages of reperfusion.

The presence of these two forms of complex I results in unusual kinetic parameters when mitochondrial preparations catalyse the inhibitor-sensitive NADH:Q oxidoreductase reaction. Depending on the preparation “history” (i.e. thermal treatment or preincubation with substrates) the initial NADH oxidation proceeds with a lag-phase (Fig. 1A) [7]. However, the final steady-state rate is linear and depends on assay conditions and on the amount of added enzyme. Although this behaviour was originally observed many years ago [7–9], it was characterised in further detail more than 30 years later and referred to as the A/D transition (see [6,10] for reviews).

The occurrence of the A/D transition was initially demonstrated for bovine complex I in preparations of heart submitochondrial particles (SMP) [11] and enzyme isolated by the Hatefi protocol [12]. Recently, it was also shown by Hirst's group with purified bovine enzyme reconstituted in proteoliposomes [13]. The enzyme spontaneously converts to the D-form after exposure of the idle preparations of mitochondria [14], SMP [11] or purified complex I [12,13] to elevated, but physiological temperatures (> 30 °C) in the absence of substrate (when catalytic turnover cannot occur). Such preparations show a considerable lag-phase during continuous assay of the NADH:ubiquinone oxidoreductase reaction (Fig. 1A, D^SH^). Addition of a small (5–10 μM) pulse of NADH before the assay results in activation of the enzyme during one or several slow turnovers when NADH is oxidised and ubiquinone is reduced. Pre-treatment with NADH completely eliminates the lag-phase, the enzyme then becomes fully active and catalyses the oxidation of NADH at a linear rate (Fig. 1A, A) [11,12]. In the presence of divalent cations (Mg^2 +^, Ca^2 +^) or at alkaline pH, the turnover-dependent activation takes longer and the lag phase is more pronounced (Fig. 1A, D^SH^). Divalent cations have no effect on the activity of the A-form.

Most of our knowledge on the kinetics of the A/D transition originated from membrane or purified preparations of the enzyme. It is likely that in highly metabolising tissues like heart, muscle and brain, at oxygen concentrations of 5–30 μM [15–17] most of complex I is in the A-form (Fig. 1B). However, under conditions in which respiration rate is decreased, the redox centres of the respiratory chain are in a reduced state due to the slowing of the cytochrome *c* oxidase. As a result, the turnover of complex I becomes restricted because of the lack of the electron acceptor ubiquinone. Therefore, the enzyme would be readily converted into the D-form (Fig. 1B, A→D transition) depending on the presence of natural effectors of the transition and overall rate of respiration [18].

The kinetics of the pseudoreversible A/D transition has been described in mammalian [11] and other eukaryotic complex I [19] and reviewed elsewhere [6,10]. However, the structural differences between the two conformations and the physiological role of this process were recently studied in more detail by our group and others. In this review we aim to summarise current knowledge on the A/D transition of mitochondrial complex I.

## 2 Differences between the A and the D-forms

### 2.1 Diagnostic tests

Based on (i) the inhibitory effect of alkaline pH and divalent cations on the activation rate and (ii) on the sensitivity of the D-form to SH-reagents, there are currently only two diagnostic tests for the estimation of the A- and D-form fractions in preparations of complex I. The content of both forms can be estimated by measuring the NADH oxidase activity of the untreated preparation in conditions arresting the activation (the A-fraction only). This should be compared with the same sample in the same measuring conditions after complex I is fully activated (total enzyme, A + D fractions together). Preparations of intact mitochondria require permeabilisation or inner membrane disruption prior to measurement in order that the active site of complex I is available for NADH [20,21].

(i) A convenient method for the determination of the A/D ratio in membrane preparations of complex I is based on the fact that at alkaline pH (> 8.5) and in the presence of divalent cations (1–5 mM Ca^2 +^ or Mg^2 +^) the rate of the D→A transition is decreased by several orders of magnitude [22]. In these conditions, the initial rate of NADH oxidase or NADH:Q oxidoreductase accurately corresponds to the activity attributable only to the A-form, since the activation time of the D-form is significantly longer than the time of the assay. The total amount of enzyme (A + D) can be estimated after full activation of complex I by preincubation of the sample with a small (5–10 μM) pulse of NADH at neutral pH prior to addition of an excess of the alkaline (pH 8.5–8.8) measuring medium containing 150 μM NADH and 1–5 mM Ca^2 +^ or Mg^2 +^ [23,24].
(ii) Since the chemical modification of the D-form results in irreversible arrest of enzyme activation, the percentage of the A-form only can be estimated after treatment of the preparation with SH-reagents, such as NEM or DTNB. The residual NEM-insensitive NADH oxidase or NADH:Q oxidoreductase activity would correspond to only the A-form of complex I. The total enzymatic activity (A + D) of the preparation can be measured after activation of the enzyme with a pulse of NADH followed by the addition of NEM.

The inhibitory effect of SH-reagents on the D-form of complex I in bovine heart SMP can be assessed by NADH oxidase or NADH:Q oxidoreductase activity which are usually affected to a similar extent [12,22,25,26]. This indicates a direct effect on complex I and not on downstream components of the respiratory chain. However, the optimal concentration of NEM (0.1–1 mM) should be determined before using mitochondrial membranes or isolated enzyme from other species, permeabilised mammalian cells and proteins extracted from blue native gel. Furthermore, the oxidation of NADH by artificial acceptors, such as hexaammineruthenium (HAR) [27], ferricyanide [28] or tetrazolium, routinely used for determination of in-gel activity of complex I [29], is not affected by the A/D transition [12,25] and therefore does not depend on the modification of the D-form by SH-reagents.

Given necessary optimisation, these two methods can be used for the estimation of the A/D content in cells, muscle fibres or tissue slices. After anoxic incubation or conditions of metabolic or chemical hypoxia [5,30–32], the preparation can be treated with a membrane permeable SH-reagent, such as NEM followed by plasma membrane permeabilisation with digitonin or saponin. The channel-forming antibiotic alamethicin can then be used to permeabilise the inner mitochondrial membrane to exogenously-added NADH [18,21]. The NEM-treatment could potentially inactivate any of the D-form of complex I present in mitochondria. Therefore, adding exogenous NADH and estimating rotenone-sensitive NADH oxidase activity will give a measure of only the A-form present in the preparation at the time of NEM-treatment. Provided all the necessary controls such as a test for completeness of membrane permeabilisation and succinate oxidase activity measurements are carried out, the time course of de-activation of complex I in particular conditions can be determined.

The A/D transition should be taken into account when measuring complex I-dependent activities in crude mitochondrial membrane preparations isolated from tissues obtained from animal models. It is likely that any chemical treatment/pharmacological intervention affecting oxygen metabolism or animal models of oxygen deprivation (global ischaemia, local infarction or stroke) would result in a significant shift in the A/D equilibrium. Depending on the degree of de-activation of the enzyme in situ and the particular conditions of mitochondrial membrane isolation, a significant fraction of the enzyme is expected to be in the D-form in the final preparation. It should be stressed that when using a medium at alkaline pH or/and supplemented with Mg^2 +^ or Ca^2 +^ for measuring complex I activity [33–37] the turnover-dependent re-activation (D→A transition) may take a significant amount of time. This could look like an apparent inhibition of the complex I-dependent reaction if the assay is started with a partially de-activated enzyme. Using conventional complex I assays in those conditions, the full enzyme activity could not be regained and might be underestimated if the reaction time is short. Special care should be taken when microplate reader spectrophotometers with preprogrammed protocols with restricted assay time are used since the phenomenon of the re-activation can be easily overlooked.

### 2.2 Fatty acid and divalent cation effects

The conversion from the D- to the A-form of complex I was shown to be inhibited by either an increase in pH or by the presence of divalent cations (Fig. 1A, D^SH^) [11,22]. However, the NADH oxidation catalysed by the A-form is almost insensitive to the presence of Ca^2 +^ or Mg^2 +^. Divalent cations possibly neutralise negative lipid charges at the membrane surface and therefore affect the process of conformational changes during re-activation. The inhibitory effect of divalent cations on the activation was found in the following order: Ni^2 +^ > Co^2 +^ > Mn^2 +^ > Ca^2 +^ ≈ Mg^2 +^ > Ba^2 +^. It should be stressed, that nickel or cobalt ions are hypoxia-mimetics and widely used to induce HIF-1α stabilisation in studies of the cellular oxygen sensing mechanism [38]. It cannot be excluded that the effect of cobalt on the A/D equilibrium can partially contribute to the cellular hypoxic response during experimentally observed HIF-1α stabilisation in various cell lines.

Free fatty acids not only affect the NADH oxidase and NADH:Q oxidoreductase activities of complex I [39–41] but also influence the dynamics of the A/D transition [42,43]. Palmitate was shown to dramatically increase the rate of spontaneous de-activation resulting in a partially irreversible inactivation of complex I at 37 °C along with a decreased rate of the D to A conversion [42]. The inhibitory effect of palmitate on the D→A transition is strongly cooperative indicating that the acting species might be a fatty acid dimer [42].

Recently the effect of Ca^2 +^ on the D→A transition was re-evaluated using pig brain SMP [43]. The inhibition of the turnover dependent D→A transition was found to be due to the combined action of free fatty acids and divalent cations. Thus Ca^2 +^ drastically potentiates the effect of free fatty acids possibly due to the formation of an ion-pair that would increase fatty acid concentration in the membrane, aggravating its effect on complex I [43].

### 2.3 The A/D transition among different species

The proton-translocating NADH:ubiquinone oxidoreductase is found in many different organisms from bacteria to mammals. These enzymes share basic catalytic properties including similar turnover number and apparent affinity constant for substrates and inhibitors [3,6,20,44,45]. As mentioned in the Introduction, the ability to switch from the A to the D state was originally observed in bovine heart mitochondria [7,46] (see [6] for historical review). Complex I is present in various organisms and its subunit composition may vary from 14 to 44, with the 14 subunit homologue in bacteria representing the smallest functional catalytic unit. Table 1 summarises the data available for complex I from various sources, but mainly emphasises the lack of information regarding the A/D transition in different organisms. Until now, the only organisms in which an A/D transition has been observed are vertebrates and fungi. The existence of the A/D transition was never tested for the plant enzyme. Complex I from the non-vertebrate organisms tested does not display an A/D transition although the enzyme possibly contains numerous accessory subunits as expected for eukaryotes. Moreover, bacterial complex I exists only in the SH-reagent insensitive form. Therefore it is likely to correspond to the A-state despite the presence of a few additional subunits as recently demonstrated for *Paracoccus denitrificans* [47] and *Thermus thermophilus* [48]. It is thus possible that the presence of certain accessory subunit(s) correlates with the A/D transition ability of the enzyme. Systematic comparison of the subunit composition between species could reveal key structural difference(s) that correlate with the ability of complex I to undergo de-activation/activation. Among the vertebrate organisms displaying the A/D transition, notable differences in the apparent activation energy determined using single transition state Arrhenius equation have been observed. Cold-blooded animals and fungi display an A/D transition with relatively low apparent activation energy, whereas warm-blooded ones are characterised by high apparent activation energy (Table 1).

### 2.4 Possible mechanism of A/D transition and subunits involved

The electron transfer from NADH to ubiquinone catalysed by complex I is coupled to the translocation of four protons across the membrane as measured experimentally [49,50] and recently supported by structural studies [51]. A conformation-driven coupling mechanism was proposed for complex I many years ago [52–55] and recently confirmed by Sazanov's group for the prokaryotic enzyme [51,56]. However the precise mechanism of energy transduction requires further elucidation. Extensive studies using cross-linking [57–61] or limited proteolysis [52,62] have been carried out on both bacterial and mammalian complex I. Even though the bacterial complex I does not display the A/D transition, the analysis of cross-linking products found in the presence/absence of substrates gives interesting information on the part(s) of the enzyme that display a degree of flexibility. This region could also be involved in the A/D transition of the enzyme from higher eukaryotes since activation (A→D transition) occurs as a result of a single slow catalytic turnover.

In bovine and bacterial complex I (*Escherichia coli* and *Thermus thermophilus*), in the presence of NAD(P)H, the quantity of cross-linked products between the hydrophilic subunits was significantly reduced and susceptibility to tryptic degradation was increased [59–61]. These observations suggested a change in the conformation of complex I upon reduction. This hypothesis was also supported by a single particle analysis on complex I from *E. coli* in the presence of NADH or NAD^+^ [63].

In the *E. coli* enzyme, the subunit NuoB (PSST in the bovine complex I) was shown to be the most mobile, moving away from NuoI (TYKY) in the presence of NAD(P)H. The NuoB subunit moves closer to other subunits, potentially NuoG (75 kDa) and hydrophobic subunits such as NuoA (ND3) or NuoH (ND1) [61]. NuoB (PSST) is known to coordinate the terminal FeS cluster N2 [64,65] and is located at the interface between the membrane and soluble domains of the enzyme, where the quinone binding pocket is found. Kinetic analysis suggested that NADH binding at the hydrophilic part and reduction of the enzyme redox centres led to a change in the conformation of the quinone binding site upon reduction [66].

Taken together, these studies clearly showed a rearrangement of the subunits around the quinone binding pocket after the binding of substrate and inhibitors in the bacterial and mammalian enzyme. However, the changes in the enzyme structure are probably relatively small, explaining results found with zero-length cross-linkers. Indeed, the use of cross-linker reagents with a spacer arm longer than 10 Å is unlikely to unveil intramolecular differences [61].

The first observation of a possible structural difference between the A- and D-forms of mitochondrial complex I was found by Vinogradov's group [67]. Using *N*-ethylmaleimide and its fluorescent derivative, they were able to show that a subunit of around 15 kDa was modified by the SH-reagent only in the D-form of the enzyme. The residue differentially labelled in the D-form of complex I only was further identified by mass spectrometry as the Cys-39 of the ND3 subunit [68]. The Cys-39 is located in the matrix hydrophilic loop connecting the second and third transmembrane helices [51,68,69]. This region is an important mutation hot spot for complex I deficiencies. Mutations in this region of ND3 lead to various mitochondrial encephalopathies indicating the importance of this loop for complex I function or regulation [70–73].

The ND3 subunit is known to participate in the formation of the quinone reaction chamber, along with ND1, 49 kDa (Nqo4 in *T. thermophilus*) and PSST (Nqo6) [51]. Changes in this region during the A/D transition were recently demonstrated via two different approaches in our group. Cross-linking studies and fluorescent labelling followed by a DIGE-like analysis were implemented to gain a better understanding of the conformational differences between the A- and the D-forms of complex I in bovine heart SMP.

To study the potential conformational changes in the A/D transition of complex I, a 6.8 Å —SH/—NH_2_ heterobifunctional cleavable cross-linker (SPDP) was used [74]. In the D-form of complex I, a cross-linked product between the ND3 subunit and the accessory subunit 39 kDa (NDUFA9) was observed [74]. This cross-linked product was absent when complex I was in the A-form (i.e. pretreated with NADH). Cross-linkers with the same reactivity but a longer arm (9.4 Å or 15.7 Å) failed to produce clear cross-linked products, despite inhibiting the re-activation of the D-form of the enzyme (A. Birch, M. Ciano unpublished results). These data suggested that the 39 kDa subunit is located near ND3, close to the quinone binding site.

Using fluorescent lysine specific reagents and a DIGE-like approach, we recently demonstrated the differential exposure of three different subunits depending on the conformation of complex I in SMP [75]. The A- and D-forms of complex I were labelled either with cyanine fluorescent Cy3- or Cy5-NHS esters before separation of complex I subunits by BN/double SDS-PAGE. As expected, the ND3 subunit was found to be more exposed in the D-form, which corroborated the earlier observation [68]. Two additional polypeptides were found more labelled in the D-form, namely the ND1 and the 39 kDa subunits. Together with our cross-linking studies, these results allowed us to conclude that the proximity of the ND3 and 39 kDa subunits in the D-form was not only due to the changed position of the hydrophilic loop of ND3, but also to a concerted rearrangement of these two subunits.

For the first time, ND1 was found associated to structural rearrangements upon de-activation of the enzyme. These three subunits are located at the junction between the hydrophilic and membrane arms of complex I and ND1 and ND3 are directly involved in the formation of the quinone binding pocket [51]. The small number of subunits found differentially exposed suggests rather discrete conformational changes located at the region of the quinone chamber. Interestingly, these results confirm the relative flexibility of complex I in that region, as observed on the bacterial enzyme in the presence or absence of NAD(P)H [61].

The junction region (Q-module) between the proton pumping (P-module) and hydrophilic module that operates the electron transfer from NADH to the terminal N2 FeS cluster (N-module) probably displays a high degree of flexibility [1]. The ability of this part of the enzyme to undergo conformational rearrangements would then not only be crucial for energy transduction, as previously suggested [64–66], but also for the process of conformational changes during the A/D transition. Therefore, de-activation of complex I would lead to the blockade of terminal electron transfer from N2 to ubiquinone due to a structural rearrangement of two crucial membrane subunits, ND1 and ND3. This may occur due to the change in position of the membrane helices of both ND3 and ND1, preventing the entrance of the ubiquinone molecule through the narrow path from the membrane [76,77]. Equally the electron transfer blockade may be due to the alteration in the tight sealing of the quinone binding chamber close to N2 after the movement of the ND3 hydrophilic loop and ND1. The precise mechanism of complex I deactivation remains to be answered.

Rotenone is a quinone-like specific inhibitor of complex I. SMP containing either the A- or the D-form of the enzyme were subjected to incubation with rotenone, the inhibitor was washed off and the content of both forms was determined. This resulted in a partial protection of the A-form against de-activation but also converted the D-form to an A-form-like conformation [78]. It has been suggested that rotenone could act as a clamp maintaining the enzyme in the A-conformation or inducing conformational changes in the D-form similar to the catalytic transitory state so that the enzyme–inhibitor complex reactivates without a turnover [78]. Rotenone also prevents the formation of cross-linked products between hydrophilic subunits, suggesting that this inhibitor could ‘lock’ complex I in a state similar to the conformation of the reduced enzyme [57].

It would then be of strong interest to perform cross-linking studies in both hydrophilic and hydrophobic parts of the mammalian complex I depending on the conformation of complex I. It is tempting to speculate that the structural rearrangement leading to the A-state might be similar to the one observed on the bacterial enzyme in the presence of NADH [61]. These conformational changes could lead to a significant modification of the shape of the quinone binding pocket, resulting in a binding and/or stabilising effect. Therefore, the A/D transition of the mammalian enzyme could have been acquired as an evolutionary process, along with the appearance of some of the accessory subunits. As suggested previously [79,80], accessory subunits surrounding the core of the enzyme may serve as a protective layer preventing access of molecular oxygen to the electron-transfer pathway of complex I. If damaged, these subunits would then simply be replaced overcoming the energy required for re-synthesis and re-assembly of the entire enzyme [80].

### 2.5 The A/D transition and supercomplexes

As demonstrated by extensive studies in Schagger's laboratory [81,82] and as observed by electron microscopy [83–86], complexes of the mitochondrial respiratory chain can be organised together into supercomplexes with various stoichiometry. These complexes are referred to as respirasomes when they contain complexes I, III and IV as they retain the ability to transfer electrons from NADH to oxygen after isolation with native electrophoresis [87–89]. It has also been suggested that this association could facilitate electron transport through the respiratory chain and prevent ROS formation [89].

The estimated apparent energy of the spontaneous A→D transition for bovine heart mitochondrial complex I is 270 kJ/mol [11]. This would suggest rather significant changes in the enzyme conformation in the membrane upon de-activation. This change could potentially alter the supramolecular organisation of supercomplexes, if present in situ. The 3D reconstruction of the bovine heart mitochondrial S_1_ supercomplex (I_1_ + III_2_ + IV_1_) shows that the complex III dimer sits in the arc of the bent membrane part of complex I so that the ubiquinone binding sites of both complexes face each other [84]. The hydrophilic loop of ND3 containing Cys-39 is located exactly in this area. Therefore, if the D-form is associated with other respiratory complexes this residue could be enclosed and inaccessible to modification by natural effectors such as ROS [24] or nitrosothiols [90]. However, using bovine SMP containing the A- or the D-form of complex I we did not find any difference either in supercomplex profiles on BN and hrCN-PAGE or any effect on supercomplex formation and on the degree of exposure of the Cys-39 of ND3 subunit in the D-form [75]. These results were further indirectly confirmed with the use of cross-linkers (EEDQ and EMCS, respectively zero-length and 9.4 Å cross-linkers). The A- or the D-form of complex I in SMP were always found cross-linked to complex IV. These results suggest a close association of complexes I and IV independently of complex I conformation (Amanda Birch, unpublished data).

## 3 Physiological role of the A/D transition

The physiological role of the A/D transition is still under discussion. As shown previously, in tissues ex vivo [23,24], a significant fraction of complex I (5–15%) was found to be in the D-form at physiological oxygen concentrations. As suggested previously, this indicates that part of the energy released during steady-state NADH oxidation in situ is used to maintain the catalytically competent A-form of the enzyme [6]. Maintenance of a fraction of complex I in the D-form would allow fast responses to changes in conditions such as reductive stress, increased ATP demand and changes in oxygen availability by analogy with the well known excess capacity phenomenon of the cytochrome *c* oxidase [91–93]. Therefore, the A/D transition could be considered as a mechanism for the fine tuning of enzyme activity [10,19,24,25]. In non-physiological conditions, i.e. ischemia/reperfusion, this fine tuning would protect not only complex I but also the whole respiratory chain from oxidative damage following reperfusion.

### 3.1 The A/D balance at limited oxygen concentration

In conditions of limited oxygen concentration (i.e. ischaemia, chemical or metabolic hypoxia [30,32]) mitochondrial redox components that are usually oxidised undergo complete reduction due to the slowing of the cytochrome *c* oxidase. Complex I turnover becomes restricted by the lack of electron acceptor ubiquinone. Accumulation of reduced quinone then not only decreases the availability of substrate for the enzyme, but also inhibits the physiological oxidoreductase activity of the enzyme [94,95]. In such situations, the steady-state equilibrium in the A↔D reaction is shifted to the right and complex I readily converted into the D-form in minutes [23,24] (Fig. 1B, A→D transition).

In the Langendorff rat heart and mouse infarction models, oxygen deprivation is shown to initiate progressive accumulation of the D-form enzyme [23,24]. The rate of complex I de-activation after cardiac arrest is much faster in brain compared to heart and may be due to the different lipid composition of the inner mitochondrial membrane. This might also explain the greater vulnerability of brain function to oxygen deprivation [69].

A potential Na^+^/H^+^ antiporter activity was demonstrated for the D-form of the bovine enzyme on proteoliposome-reconstituted complex I and SMP [13]. It was suggested that the Na^+^/H^+^ exchange catalysed by the D-form accumulated during the ischaemic period can contribute to the protection of mitochondrial ion balance [13]. The potential antiporter activity of the enzyme could be important for the maintenance of the ion-balance in mitochondria during ischaemia, contributing to the regulation of Na^+^ concentration in the cytosol [96].

### 3.2 The A/D transition during reoxygenation

Cardiac reperfusion following ischemia/anoxia results in the return of complex I A/D equilibrium to its initial level (A/D ratio ≈ 9–7) indicating that reintroduction of oxygen causes re-activation of the D-form of the enzyme [23,24]. Therefore, the A/D transition in cardiac tissue takes place in situ during ischaemia and this reversible process would play a role in tissue response to oxygen deprivation (Fig. 1B, A↔D).

As shown previously, chemical or pharmacological inhibition of complex I during ischaemic incubation would protect mitochondria from damage after reperfusion [90,97–101]. The presence of pyridaben, an inhibitor of complex I, decreased lesion size in brain and reduced oxidative damage during hypoxia/ischaemia in neonatal mice [102]. The presence of another complex I inhibitor, amytal, in the ischaemia/reperfusion (I/R) model decreased the amplitude of the free radical's EPR signal and lessened lipid peroxidation [103].

Following ischemia/anoxia, reoxygenation may initiate abnormal bursts of NAD(P)H oxidation, ROS generation and the formation of other reactive metabolites capable of damaging mitochondrial constituents including complex I. The de-activation of complex I could then be a physiological mechanism to maintain a low enzymatic activity when oxygen concentration rises. The presence of most of complex I in the D-state during reintroduction of oxygen to the tissue would have two important consequences. Firstly, the slow activation of complex I during the early phase of reoxygenation would reduce the burst of respiration downstream of the enzyme. Secondly, it would delay rapid formation of potentially ROS-promoting tightly bound ubisemiquinones in complex I [104–107] until the oxygen concentration decreases to the normoxic steady-state level due to the activity of the cytochrome *c* oxidase. Therefore, the lag-phase in complex I activity, although probably very short in situ, would decrease the generation of ROS by the mitochondrial respiratory chain and lessen the oxidative damage. It is possible that the reversible de-activation of complex I in the absence of oxygen may function as a protective valve for complex I electron transfer pathway upon tissue reoxygenation.

Another important consideration is the effect of de-activation on reverse electron transfer. In the presence of membrane potential, ubiquinol is able to reduce redox-centres of complex I such as all iron sulphur clusters and flavin, so that NAD^+^ can be reduced to NADH [108,109]. In vitro, the reverse electron transfer is accompanied by increased H_2_O_2_ production which is prevented by complex I inhibitors [102,110–113]. The reduced flavin of complex I is likely to be the main source of superoxide, which is sequentially converted to H_2_O_2_ [114,115]. The D-form of the enzyme is unable to catalyse the reverse transfer of electrons and therefore de-activation could act as a safety valve preventing reduction of the enzyme from downstream [11]. Since in post-hypoxic conditions the entire pool of NAD(P)^+^ is reduced it is unlikely that the reverse electron transfer occurs directly from ubiquinol to the nucleotide. However, during reoxygenation, de-activation can temporarily prevent reduction of FMN in a fraction of the enzyme. Since respiratory chain complexes are distributed heterogeneously in the inner mitochondrial membrane along the cristae and therefore can be exposed to different redox conditions (NAD(P)H/NAD(P)^+^ ratio) and/or potential [116].

A critical consequence of I/R process is oxidative stress and therefore special endogenous mechanisms should have evolved in order to ameliorate the damage. This damage is unlikely to be directly due to a decrease in the respiratory chain activity and ATP production per se, but due to oxidative stress following reintroduction of oxygen in tissues in the post-ischaemic overreduced state [5]. The ischaemic de-activation of complex I might initially act as an intrinsic protective mechanism against overproduction of ROS and provide a safe way to recover cellular bioenergetic function to the normal state after reoxygenation.

### 3.3 Covalent modifications

Covalent modification of thiol groups via reversible glutathionylation, oxidation, nitrosation [117] or the formation of Michael adducts [118] are important factors in the regulation of enzyme activity and cellular signalling. Exposure of Cys-39 of the ND3 subunit upon deactivation is one of the main conformational changes identified in complex I to date. It is thought that this could be important in several pathological scenarios [18,67,68,90].

Following the discovery that the glutathionylation of some complex I subunits increased superoxide production by the enzyme [119] it was tempting to speculate that the modification of the D-form of complex I by the GSH/GSSG couple could regulate the enzyme's activity. However, the D-form of the enzyme was found to be insensitive to reduced or oxidised glutathione in vitro [25,26]. Modification of cysteine thiols in situ, however, depends on particular conditions including: redox environment, GSH/GSSG ratio, hydrophobicity surrounding the target thiol, pH, ion composition, and the activity of enzymes mediating the glutathionylation reaction: glutathione-S-transferases and the glutaredoxin system [117,120,121].

Recently, complex I conformation-specific thiol modifications were identified in mitochondrial membranes isolated from normoxic and ischaemic mouse heart [24]. Exogenous H_2_O_2_ and superoxide were found to inhibit only the D-form of complex I [24] whilst activation of the enzyme completely abolished the ROS-mediated inhibition. Cys-39 was found to be modified in complex I during treatment with H_2_O_2_. Once Cys-39 of the ND3 subunit is modified, complex I is thought to be unable to undergo the D→A conversion and catalyse the physiological NADH:ubiquinone reaction. Thus, Cys-39 may be an early mitochondrial target for oxidative/nitrosative stress during I/R.

Inhibition of complex I-mediated respiration was demonstrated in cells after they were incubated with activated macrophages [122]. This inhibition of complex I activity was found to be due to nitric oxide (NO) [123]. Prolonged exposure to a high concentration of NO was found to result in persistent inhibition of complex I in cells [124–126], which was later confirmed by other groups [127–131] and attributed to nitrosation of critical thiol(s). Interestingly both the A- and D-forms of complex I were found insensitive to direct treatment with NO in bovine SMP. However, the re-activation of the D-form was inhibited by nitrosothiols and peroxynitrite whilst the A-form was insensitive to such treatment [25].

This was recapitulated in HEK cells producing endogenous NO [18]. Due to the inhibitory action on the cytochrome *c* oxidase [92,132,133], NO induced the de-activation of complex I in hypoxia leading to the exposure of Cys-39 of the ND3 subunit. This thiol group is then susceptible to covalent modification by NO-metabolites. It is then expected that modification of this SH-group would result in inhibition of cellular respiration. Indeed, this has been observed in HEK293-tet-iNOS cells transfected with NO-synthase [134] and by measuring the autofluorescence of matrix nucleotides in intact cells with confocal microscopy [18]. This site of complex I nitrosation was identified in Murphy's laboratory using the mitochondria-targeted S-nitrosothiol ‘MitoSNO’ [90]. MitoSNO was found to nitrosate Cys-39 of the ND3 subunit and inhibit complex I after but not before ischaemia in an intact heart model of cardiac I/R [90]. S-nitrosation of Cys-39 of the ND3 subunit in hypoxic conditions was also found to slow reactivation of mitochondrial respiration during reperfusion. This decreased reactivation rate of the enzyme was found to be concomitant with a decrease in ROS production, oxidative damage and tissue necrosis [90]. Exposure of Cys 39 in the D-form of the enzyme under ischaemic conditions may act as the switch preventing excess ROS production by the enzyme during reperfusion. Therefore, slowing the rapid reactivation of complex I by modification of Cys-39 of ND3 may represent a novel therapeutic strategy that could be important in various pathological and physiological scenarios such as I/R, chronic inflammation, neurodegeneration or mitochondrially inherited diseases.

The kinetics of the A/D transition can be significantly affected in different conditions but the degree of modification of the D-form by ROS or NO metabolites in situ is not clear at present. S-nitrosation of the ND3 subunit is probably reversible via reduction by glutathione and thioredoxin systems and may be protective (Fig. 1B, D^SNO^) [90]. Nitration or oxidation of thiol groups (Fig. 1B, D^S⁎^), however, is irreversible at the timescale of I/R. Prolonged exposure of the D-form of the enzyme to low steady-state levels of endogenous SH-reactive molecules such as *S*-nitrosoglutathione, peroxynitrite and ROS would lead to modification of a certain fraction of the enzyme. Depending on the nature of the covalent modification, the fraction of modified enzyme would gradually increase subject to the presence of the effector and activity of the thiol-regenerating system. Complex I has a high degree of flux control over oxidative phosphorylation [135–137] and therefore arresting the re-activation of even a small fraction of the enzyme may have a significant effect on the matrix NAD^+^/NADH ratio and also the membrane potential and ATP production. Depending on the magnitude and time-frame of the process, the degree of oxidative damage and the course of recovery in ischaemic tissues after reperfusion could be significantly affected.

## 4 Conclusion

The transition of mitochondrial complex I from the A- to the D-state is a unique feature of some eukaryotic enzymes, including mammals. The mechanism of the A/D transition of the enzyme is far from being understood, however one of the accessory subunit, 39 kDa (NDUFA9) and two mitochondrially encoded subunits, ND3 and ND1, all located in the area of the quinone binding site are involved in conformational rearrangements. It is unlikely that the A/D transition is induced by changes in the respiratory chain supercomplex assembly. The driving force of the A/D transition, the mechanism of the electron transfer blockage in the D-form and other potential accessory subunits involved remain to be clarified.

Results from our and other laboratories suggest that accumulation of the D-form of complex I occurs in mitochondria-rich tissues during oxygen deprivation. The rate of accumulation of the D-form of the enzyme during ischaemia can be modulated by elevated temperature, the presence of free fatty acids and divalent cations. This would affect the redox state of cofactors and reactive centres upstream and downstream of the enzyme. The A/D transition may represent a natural mechanism providing a fast mitochondrial response to oxygen deprivation. Deceleration in the activity of the respiratory chain due to the slow activation of the D-form during the initial phase of reoxygenation would prevent mitochondrial generation of ROS, decreasing oxidative damage. However, the cysteine-39 of the ND3 subunit, exposed in the D-form, is susceptible to covalent modification by ROS and NO metabolites. Accumulated D-form could react with natural effectors in mitochondria or with pharmacological agents during periods of hypoxia or reoxygenation. This would modulate the process of reactivation of the enzyme and therefore determine the outcome of the I/R event.

## Figures and Tables

**Table 1:** Subunit composition and A/D transition of complex I from different organisms.

Species	A/D transition	Number of subunits	MW (kDa)	E_A_ for de-activation (kJ/mol)	E_A_ for activation (kJ/mol)
Eukaryote					
Vertebrate					
*Bos taurus* (bovine)	Yes^a^[11]	44 [138,139]	980 [138,139]	270 [11]	170 [11]
*Rattus norvegicus* (rat)	Yes^a^[14,140]	N.D.	N.D.	N.D.	N.D.
*Gallus gallus* (chicken)	Yes^a^[19]	N.D.	N.D.	N.D.	N.D.
*Sus scrofa domesticus* (pig)	Yesa,b[141]	N.D.	N.D.	N.D.	N.D.
*Rana catesbeiana* (frog)	Yesc,d[19]	N.D.	N.D.	151 [19]	66 [19]
*Cyprinus caprio* (carp)	Yesc,d[19]	N.D.	N.D.	204 [19]	67 [19]
Non-vertebrate					
*Lumbricus terrestris* (earthworm)	No [19]	N.D.	N.D.	–	–
*Acheta domesticus* (cricket)	No [19]	N.D.	N.D.	–	–
*Homarus americanus* (lobster)	No [19]	N.D.	N.D.	–	–
Fungi					
*Yarrowia lipolytica*	Yes [19]	≥ 40 [142]	≥ 946.5 [142]	≪ 270 [19]	N.D.
*Neurospora crassa*	Yes [143]	39 [144]	> 700 [144]	≪ 270 [19]	N.D.
Plant					
*Arabidopsis thaliana*	N.D.	~ 30 [145]	~ 1000 [146]	N.D.	N.D.
Algae					
*Chlamydomonas reinhardtii*	N.D.	42 [147]	~ 970 [147]	N.D.	N.D.
Prokaryote					
Bacteria					
*Escherichia coli*	No [19]	14 [148]	~ 550 [148]	–	–
*Paracoccus denitrificans*	No [149]	17 [47,150]	~ 530 [47,150]	–	–
*Rhodobacter capsulatus*	No^e^	14 [151]	~ 550 [151]	–	–
*Thermus thermophilus*	No [19]	16 [48,51]	~ 536 [48,51]	–	–

N.D.: not determined.

a Heart.

b Brain.

c Skeletal muscle.

d Liver.

e A. Galkin unpublished data.
